# O.4.5-4 What do young people want in a sport program: formative evaluation using Concept Mapping

**DOI:** 10.1093/eurpub/ckad133.212

**Published:** 2023-09-11

**Authors:** Alex Donaldson, Kiera Staley, Erica Randle, Matthew Nicholson

**Affiliations:** La Trobe University, Australia; La Trobe University, Australia; La Trobe University, Australia; Monash University Malaysia; a.donaldson@latrobe.edu.au

## Abstract

**Purpose:**

Engaging young people in sport and physical activity (PA) requires awareness of their preferences and priorities. This study provided less active young people (14–17 years of age) with an opportunity to articulate their perspective on an ideal sport program.

**Methods:**

We used online concept mapping, involving qualitative data collection and quantitative data analysis. Forty-eight less active young people in regional Victoria, Australia brainstormed 65 relevant, unique and single-focus statements in response to the prompt: *The features of a sport program that I would really like to participate in are….* Twenty-four participants then sorted these statements into groups based on similarity of meaning, and 37 participants rated each statement on 5 point Likert scales for its *importance* (1 = completely unimportant; 5 = very important) in a program and how *common* (1 = never; 5 = always) it is in the programs they participate in. We analysed sorting data using multi-dimensional scaling and hierarchical cluster analysis, and rating data using descriptive statistics.

**Results:**

We identified a map with the following nine clusters (in order of relative mean importance ratings)—*access and convenience* (mean importance rating = 4.11 out of 5; mean common rating 3.83 out of 5); *social connection* (3.98; 3.78); *inclusion and belonging* (3.98; 3.53); *confidence* (3.86; 3.46); *design and structure* (3.85; 3.65); *skill development and goals* (3.84; 3.60); *cost and time* (3.80; 3.30); *age and gender* (3.61; 3.41); and *specific features* (3.53; 3.25)—as the most appropriate representation of the participants’ sorting data. The two single statements—both in the *inclusion and belonging* cluster—rated most important were a sport program *where you are accepted* (4.57) and *in which you do not feel judged* (4.51).

**Conclusions:**

Young people want affordable, accessible, convenient, safe, welcoming, socially engaging and inclusive sport and PA programs. They want programs to be well organised, delivered by experienced coaches with flexible content that builds their confidence, fitness and skills through challenging and fun activities. Youth sport and PA program designers can use this data to inform program development and delivery planning.

**Support/Funding Source:**

Victorian Health Promotion Foundation (VicHealth).

